# Adherence to Personal Protective Equipment Guidelines During the COVID-19 Pandemic Among Health Care Personnel in the United States

**DOI:** 10.1017/dmp.2021.12

**Published:** 2021-01-08

**Authors:** Oliver A. Darwish, Ayushi Aggarwal, Mehran Karvar, Chenhao Ma, Valentin Haug, Mengfan Wu, Dennis P. Orgill, Adriana C. Panayi

**Affiliations:** 1 California Northstate University College of Medicine, Elk Grove, CA, USA; 2 Division of Plastic Surgery, Department of Surgery, Brigham and Women’s Hospital and Harvard Medical School, Boston, MA, USA; 3 University of Maryland School of Medicine, Baltimore, MD, USA; 4 Department of Hand, Plastic and Reconstructive Surgery, Microsurgery, Burn Center, BG Trauma Center Ludwigshafen, University of Heidelberg, Ludwigshafen, Germany; 5 Department of Plastic and Cosmetic Surgery, Nanfang Hospital, Southern Medical University, Guangzhou, Guangdong, P R China

**Keywords:** adherence, coronavirus, COVID, COVID-19, PPE, safety

## Abstract

**Objectives:**

Protecting frontline health care workers with personal protective equipment (PPE) is critical during the coronavirus disease (COVID-19) pandemic. Through an online survey, we demonstrated variable adherence to the Centers for Disease Control and Prevention (CDC) PPE guidelines among health care personnel (HCP).

**Methods:**

CDC guidelines for optimal and acceptable PPE usage in common situations faced by frontline health care workers were referenced to create a short online survey. The survey was distributed to national, statewide, and local professional organizations across the United States and to HCP, using a snowball sampling technique. Responses were collected between June 15 and July 17, 2020.

**Results:**

Responses totaling 2245 were received from doctors, nurses, midwives, paramedics, and medical technicians in 44 states. Eight states with n > 20 (Arizona, California, Colorado, Louisiana, Oregon, South Carolina, Texas, and Washington) and a total of 436 responses are included in the quantitative analysis. Adherence to CDC guidelines was observed to be highest in the scenario of patient contact when COVID-19 was not suspected (86.47%) and lowest when carrying out aerosol generating procedures (AGPs) (42.47%).

**Conclusions:**

Further research is urgently needed to identify the reasons underlying variability between professions and regions to pinpoint strategies for maximizing adherence and improving the safety of HCPs.

## Introduction

A critical focus of the US response to coronavirus disease (COVID-19) is to protect nearly 18 million health care personnel (HCP).^1^ Without proven, effective treatments, use of personal protective equipment (PPE) remains key to HCP safety. To date, the Centers of Disease Control and Prevention (CDC) has provided PPE recommendations in the United States to facilitate a safer work environment.^2,3^ Through an online, anonymous survey, we collected data on adherence to CDC PPE guidelines among HCP in different US states.

## Methods

By referencing guidelines from the CDC,^2,3^ we defined the standard of optimal PPE use in 5 different clinical scenarios (Table 1). Following approval from the review board at Brigham and Women’s Hospital, an online questionnaire was designed, using REDCap (Vanderbilt University, Tennessee), and distributed among statewide health care institutions and to HCP through the snowball sampling technique. Adherence was determined by calculating the proportion of respondents who met the optimum PPE standard. Data were processed and analyzed using Microsoft Excel 365 (Microsoft, WA) and RStudio 1.2.5033 (RStudio, MA).

**Table 1.** Clinical scenarios and optimal personal protective equipment combinations

Setting	Requirements*
Communal hospital space	Face mask (or respirator)
Patient contact when COVID-19 is not suspected	Face mask (or respirator)
Patient contact when COVID-19 is suspected	Respirator (or face mask), gloves, eye protection, and isolation gown
Patient contact when COVID-19 is confirmed	Respirator (or face mask), gloves, eye protection, and isolation gown
Carrying out Aerosol Generating Procedures (AGPs)	Respirator, gloves, eye protection, and isolation gown

*Note*:

* To accommodate for PPE shortages, the CDC guidelines state that surgical masks are an acceptable alternative for patient contact when COVID-19 is suspected or confirmed when there is a shortage of respirator masks. We accommodate this as adherent to the guidelines.

## Results

**Figure 1.**
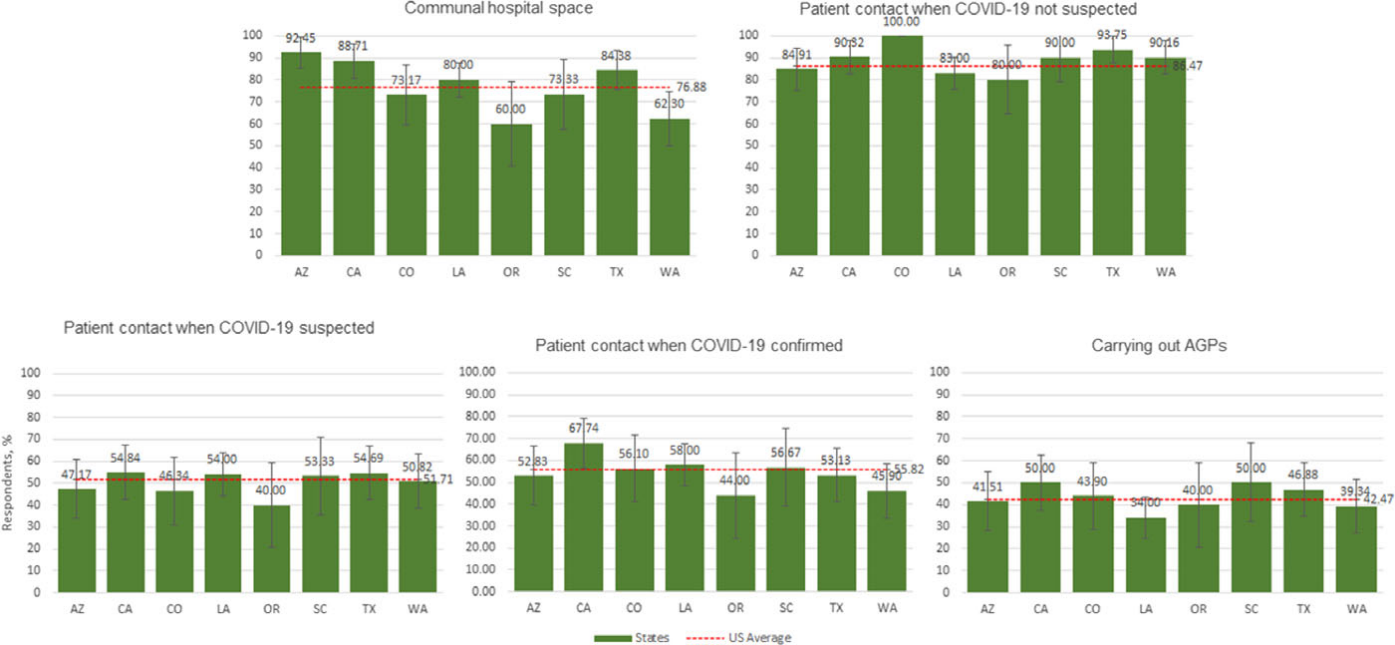
Percent adherence to optimum personal protective equipment (PPE) guidelines in 5 clinical scenarios in different states: WA, Washington; TX, Texas; SC, South Carolina; OR, Oregon; LA, Louisiana; CO, Colorado; CA, California; AZ, Arizona.

From June 15 to July 17, 2020, 2245 responses from 44 US states were collected; 436 responses from 8 states – Washington (n = 61); Texas (n = 64); South Carolina (n = 30); Oregon (n = 25); Louisiana (n = 100); Colorado (n = 41); California (n = 62); Arizona (n = 53) – are included in the quantitative analysis. Only the first 100 of 1760 responses received from Louisiana were analyzed to control for sampling bias. Respondents included nurses (57.8%), doctors (26.8%), midwives (11.5%), paramedics (1.9%), medical technicians (1.2%), and unknown/unstated (0.7%). Among different professions, doctors demonstrated greatest adherence in all scenarios except for “patient contact when COVID-19 suspected,” for which nurses demonstrated the highest adherence. The rate of adherence to optimum PPE guidelines in the 8 states included is shown in Figure 1.

## Discussion

HCP adherence to PPE guidelines has remained a key concern throughout the ongoing COVID-19 crisis, particularly because effective prophylaxis and treatments have yet to become available. Based on a prior global survey study conducted by Panayi et. al.^4^ regarding HCP adherence to the World Health Organization PPE recommendations, we investigated nationwide adherence to CDC PPE recommendations among HCP, during a time when the COVID-19 crisis has been rapidly evolving.

Among the 8 states included in this report, the highest and lowest complete adherence to 4 of 5 clinical scenarios were seen in California and Oregon, respectively. While adherence to the PPE guidelines in different clinical scenarios varied widely, our results showed average adherence in each scenario is poor (ranging from 42.47% to 86.47%). As a general trend, HCP adherence to PPE in “Patient contact when COVID-19 is not suspected” was highest, and in “Carrying out AGPs” was lowest compared with the other clinical scenarios.

Although this study provides new insights on the use of PPE among HCP, its limitations cannot be ignored. Data were included from only 8 states, which limits the generalizability of the results. Further, individual institutional guidelines can influence HCP understanding of optimum standard PPE-use in different scenarios. PPE availability is changing rapidly as state and local decision makers adapt to their situations. These data represent only 1 time frame during the course of a longer public health crisis and should be considered in this context.

## Conclusions

Factors driving low adherence rates may be PPE shortages, insufficient training, and/or a low level of awareness among HCP, as well as the inability to remain informed on rapidly evolving recommendations by the CDC or individual institutional administrations. Although our survey does not reflect possible reasons for such variability, it highlights the need to address disparities in PPE use among different HCPs and to urgently investigate the factors that may be contributing to disparities among the states. Providing effective training programs, an adequate supply of PPE, and in-training evaluation are top strategies that can be enforced by policy-makers and safety managers of health care institutions. Further research is warranted to identify which strategies may be most useful.
